# GABA Administration Ameliorates Sjogren’s Syndrome in Two Different Mouse Models

**DOI:** 10.3390/biomedicines10010129

**Published:** 2022-01-07

**Authors:** Min Song, Jide Tian, Blake Middleton, Cuong Q. Nguyen, Daniel L. Kaufman

**Affiliations:** 1 Department of Molecular and Medical Pharmacology, University of California, Los Angeles, CA 90095, USA; minsong1961@hotmail.com (M.S.); jtian@mednet.ucla.edu (J.T.); numbersix@gmail.com (B.M.); 2 Department of Oral Biology, College of Dentistry, University of Florida, Gainesville, FL 32610, USA; nguyenc@ufl.edu

**Keywords:** Sjögren’s syndrome, GABA, autoimmune disease, immunotherapy, GABA-receptor, dry eye disease, salivary gland, lacrimal gland

## Abstract

Sjögren’s syndrome (SS) is a chronic autoimmune disease characterized by lymphocytic infiltrates in the salivary and lachrymal glands resulting in oral and ocular dryness. There are no clinically approved therapies to slow the progression of SS. Immune cells possess receptors for the neurotransmitter GABA (GABA-Rs) and their activation has immunoregulatory actions. We tested whether GABA administration has potential for amelioration of SS in NOD.B10-H2^b^ and C57BL/6.NOD-Aec1Aec2 mice, two spontaneous SS models. Oral GABA treatment was initiated (1) after the development of sialadenitis but before the onset of overt symptoms, or (2) after the appearance of overt symptoms. When assessed weeks later, GABA-treated mice had greater saliva and tear production, as well as quicker times to salvia flow, in both SS mouse models. This was especially evident when GABA treatment was initiated after the onset of overt disease. This preservation of exocrine function was not accompanied by significant changes in the number or area of lymphocytic foci in the salivary or lachrymal glands of GABA-treated mice and we discuss the possible reasons for these observations. Given that GABA-treatment preserved saliva and tear production which are the most salient symptoms of SS and is safe for consumption, it may provide a new approach to help ameliorate SS.

## 1. Introduction

Sjögren’s syndrome (SS) is a complex chronic autoimmune disease characterized by immune cell infiltration into exocrine glands, particularly the salivary and lacrimal glands, which leads to a severe loss of secretory function and consequent xerostomia (dry mouth) and keratoconjunctivitis sicca (dry eyes) [1,2,3,4]. Patients with SS also experience an array of manifestations ranging from skin vasculitis to neuropathy and lymphoma. The disease affects four million Americans with over 90% of those affected being female [5]. The underlying etiology of SS is thought to be heterogeneous and remains elusive but is thought to involve abnormal salivary gland homeostasis and progressive tissue damage by infiltrating immune cells and autoantibodies. Analysis of human salivary glands and experimental mouse models of SS reveals that focal lymphocytic infiltrates are dominated by CD4^+^ T cells with B cells and macrophages increasing with disease progression [6,7,8,9,10,11,12]. Experimental evidence points to Th17, Th1, Th2, and follicular helper T (Tfh) cells as contributors to the pathogenesis of SS [12,13,14,15,16,17,18,19,20]. Additionally, several types of autoantibodies are associated with the development and progression of SS, including anti-nuclear, rheumatoid factor, anti-SSA/Ro, anti-SSB/La and anti-M3R autoantibodies [19].

Currently, treatments for SS are largely limited to ameliorating the disease’s symptoms with artificial tears and mouthwash. Application of cyclosporine and rebamipide in eye drops has had beneficial effects in a limited number of SS patients, and clinical trials with immunosuppressants failed to reach the primary endpoints of efficacy (reviewed in [19]. Research efforts in animal models of SS have identified a number of treatments with therapeutic effects, but often their safety has not been established and they have not yet reached clinical testing. Accordingly, there is an urgent need to identify additional drugs, particularly those with an established safety profile, that could help ameliorate SS.

We, and others, have shown that many types of immune cells have receptors for the neurotransmitter GABA and that the activation of these receptors generally has anti-inflammatory activities [21,22,23,24,25,26,27,28,29,30,31,32,33,34,35,36]. T cells express type A GABA-Rs (GABA_A_-Rs) [21,25,28,29,35,36,37,38] and the oral administration of GABA or the GABA_A_-R agonist homotaurine inhibits autoreactive CD4^+^ Th1 and Th17 cell as well as autoreactive CD8^+^ T cell responses, and reduces autoantibody levels, while simultaneously promoting CD4^+^ and CD8^+^ Treg responses [26,28,29,39]. The oral administration of GABA-R agonists ameliorates autoimmune disease in mouse models of type 1 diabetes (T1D), multiple sclerosis, and rheumatoid arthritis, and also limits inflammation in murine type 2 diabetes [22,28,29,35,37,40]. Recently, we have shown that GABA administration can limit the excessive inflammatory responses that cause pneumonitis and death in mice infected with a murine coronavirus [36]. Thus, GABA-R agonists can inhibit disease in different models of chronic autoimmune diseases, which have different etiologies and occur in mice with different genetic backgrounds. Human T cells and antigen-presenting cells also express GABA_A_-Rs and their activities are regulated by GABA_A_-R agonists and antagonists [25,38,41,42]. Notably, GABA inhibits secretion of IL-6, TNF, IL-17A, CXCL10/IP-10, CCL4, CCL20, and MCP-3 from anti-CD3 stimulated PBMC from T1D patients [38]. Yet, GABA does not cause lymphopenia and leukopenia and is safe for human consumption, making it a promising candidate for ameliorating inflammatory disorders.

Based on the effectiveness of GABA-R agonist-based therapies to ameliorate other models of autoimmune diseases, we hypothesized that GABA treatment could be an effective therapy for ameliorating SS. We tested this hypothesis in two different spontaneous models of SS, which occur in mice with different genetic backgrounds. NOD.B10-H2^b^ mice were derived from type 1 diabetes-prone nonobese diabetic (NOD) mice by replacing their MHC I-Ag^7^ loci with the MHC I-A^b^ from C57BL/10 mice [43]. NOD.B10-H2^b^ mice spontaneously develop exocrine inflammatory infiltrates, anti-nuclear autoantibodies and develop SS with a female predilection [43,44,45,46]. They do not develop insulitis, hyperglycemia, or T1D, which could be potential confounds in our studies [47]. Exocrine tissue from female NOD.B10-H2^b^ mice displays robust lymphocytic infiltrates in the salivary and lacrimal glands with progressive loss of saliva and tear secretion. The infiltrates become evident by three months in age, and clinical disease manifests at twenty-four weeks in age with measurable loss of saliva and tear production [43,44,45,46].

We also studied C57BL/6.NOD-Aec1Aec2 mice which are C57Bl/6 mice which carry two loci, Idd3 and Idd5 (Aec1 and Aec2), from NOD mice that are necessary and sufficient to cause a SS-like disease and are in synteny with genetic regions associated with SS in humans [48,49]. A systems biology comparison of genome-wide expression data from the salivary glands of SS patients versus that of C57BL/6.NOD-Aec1Aec2 mice identified common dysregulated biological pathways, supporting the notion that these mice provide a good model for studies of interventive therapies [50]. In these mice, leukocyte infiltration into exocrine glands begins at 8–16 weeks in age, along with an increase in salivary gland proinflammatory cytokines [13,16,48,49]. The infiltrates have a similar composition to that in the human disease, with a similar dramatic increase in Th17 cells. Notably, the elimination of IL-17 in these mice reduces sialadenitis [16]. Moreover, transcriptome analysis of their salivary and lacrimal glands at an early stage of the disease revealed the induction of IFNγ-stimulated genes [51]. After 16 weeks in age, these mice display pronounced salivary and lacrimal secretory dysfunctions.

Here, we present the results of our studies of prophylactic and interventive GABA treatment on exocrine functions in the NOD.B10-H2^b^ and C57BL/6.NOD-Aec1Aec2 mouse models of SS.

## 2. Materials and Methods

### 2.1. Mice

Female NOD.B10-H2^b^ mice (the Jackson Laboratory) and C57BL/6.NOD-Aec1Aec2 mice were studied due to their greater propensity to develop sialadenitis compared to male mice. The derivation of C57BL/6.NOD-Aec1Aec2 mice has been previously described [52]. Mice were bred and maintained under specific pathogen-free conditions with a 12-h light/dark cycle in the Division of Laboratory Animal Medicine at UCLA. They were provided food and water ad libitum. This study was carried out in accordance with the recommendations of the Guide for the Care and Use of Laboratory Animals of the National Institutes of Health. The protocols for all experiments using vertebrate animals were approved by the Animal Research Committee at UCLA (protocol #1993–2001).

### 2.2. Reagents

GABA, pilocarpine, ketamine, xylazine, and isoproterenol were purchased from Sigma-Aldrich (St. Louis, MO, USA).

### 2.3. GABA Treatment

At the indicated age, mice were randomized and given plain water or water containing GABA (at 6 or 20 mg/mL as indicated) continuously through the drinking water. The drinking water was changed every five days. Previous studies have shown that mice given GABA through their drinking water consume the same amount of food and water as mice on plain water [22,39].

### 2.4. Measurements of Saliva Production

Saliva production was measured as described [16] with slight modifications. Individual mice were weighed and lightly anesthetized by ketamine (8 mg/g of body weight)/xylazine (4 μg/g of body weight). After 5 min, tear and saliva secretion was stimulated by intraperitoneal injection with PBS containing freshly prepared pilocarpine (40 μg/100 μL) and isoproterenol (20 μg/100 μL). Immediately after stimulation, the secreted saliva was collected with a micropipette that was inserted into the oral cavity. The time to the start of saliva flow was recorded and saliva was collected for 10 min thereafter. The collected saliva from each mouse was transferred into a pre-weighed Eppendorf tube and the weight of the collected saliva was determined. The saliva flow rate was calculated as the weight of saliva collected per gram weight of the mouse.

### 2.5. Measurement of Basal Tear Production

Under light anesthesia, a phenol red thread (ZoneQuick, Ophthalmics, Pembroke, MA, USA) was held in the lateral canthus of one eye with a forceps. After 20 s, the thread was removed and the entire wet (red) portion was measured and recorded in millimeters using the scale provided in the Zone-Quick test kit. This was repeated for the other eye and these two measurements were averaged for each mouse.

### 2.6. Histological Analysis of Salivary and Lacrimal Gland Tissues

The day after collecting tear and saliva, the mice were humanely euthanized and their submandibular gland (SMG) and exorbital lacrimal gland (ELG) were excised, fixed in 10% formalin for 24 h and paraffin-embedded. The tissue sections (4 µm) were routine-stained with by H and E. Using a Nikon Eclipse 90i upright microscope fitted with a Nikon DS-Qi1Mc camera, the H and E stained sections were photo-imaged with a 4X objective lens. The number of lymphocytic infiltrate foci (defined as an aggregate of >50 monocytes) were counted in each SMG or ELG section. To determine the percent infiltrate area, the infiltrate areas and the total tissue section area of each image were measured using Nikon NIS-Element software. The data are presented as the mean percentage of infiltrate area in the SMG or ELG sections. One SMG or ELG tissue section was analyzed per mouse. All slides were coded with ID numbers and the histological assessments were performed in a blinded manner.

### 2.7. Statistical Analysis

All normally distributed data were analyzed by two-tailed Student’s *t*-test and the data are shown as the mean ± SD of each group. Non-normally distributed data are shown in boxplots with the borders of the box indicating 1st and 3rd quartile, the bolded line indicating the median, and with outliers plotted outside the whiskers. The non-normally distributed data were analyzed by Mann-Whitney U test if applicable. A *p*-value of <0.05 was considered statistically significant.

## 3. Results

### 3.1. Prophylactic GABA Treatment in NOD.B10-H2^b^ Mice

In NOD.B10-H2^b^ mice, sialadenitis becomes histologically observable beginning at about 12 weeks in age and overt clinical symptoms such as reduced saliva and tear production are apparent by 24 weeks in age [43,44,45,46]. Eighteen weeks old female NOD.B10-H2^b^ mice were randomized to continuously receive plain water or water supplemented with GABA (6 mg/mL). Fourteen weeks later, at thirty-two weeks in age, we analyzed their saliva flow and tear production.

**Figure 1:**
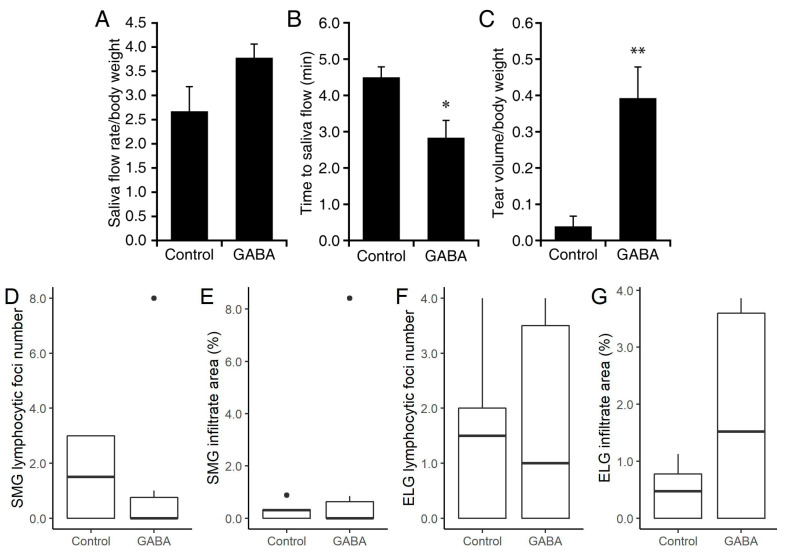
Prophylactic GABA treatment in NOD.B10-H2^b^ mice. Eighteen weeks old female NOD.B10-H2^b^ mice were placed on plain water, or GABA-containing water (6 mg/mL) for fourteen weeks. At 32 weeks in age, we analyzed their saliva flow, the time to saliva flow, and their basal tear production following isoproterenol and pilocarpine injection, as well as the number of lymphocytic foci in their SMG and ELG. (**A**) Saliva production. Data shown is mean amount of saliva ± SD collected over 15 min per gram body weight. (**B**) Time to saliva production (minutes). (**C**) Tear production. Data shown is mean ± SD of the length of thread wetting (mm) over 20 s (average of both eyes) per gram body weight at 10 min post- pilocarpine and isoproterenol injection. (**D**) Mean number ± SD of lymphocytic foci in the SMG, (**E**) mean area ± SD of infiltrates in the SMG, (**F**) mean number ± SD of foci in the ELG, and (**G**) mean area ± SD of infiltrates in the ELG of control and GABA-treated mice. All normally distributed data were analyzed by two-tailed Student’s *t*-test. Non-normally distributed data are shown in boxplots with the borders of the box indicating 1st and 3rd quartile, the bolded line indicating the median, and with outliers plotted outside the whiskers and indicated by a “●”. For all data *n* = 4 control and 6 GABA-treated mice. * *p* < 0.05 and ** *p* < 0.01.

Saliva and tear production was stimulated by isoproterenol and pilocarpine administration as per [53]. We observed that GABA-treated NOD.B10-H2^b^ mice produced an average of 41% more saliva than the NOD.B10-H2^b^ mice given plain water although this was not statistically significant (Figure 1A). The time to saliva production was significantly shorter in GABA-treated mice (*p* < 0.05, Figure 1B). Impressively, the amount of tear production was on average about 10-fold higher in the GABA-treated group vs. the plain water-treated group (*p* < 0.01, Figure 1C). Thus, prophylactic GABA treatment tended to preserve saliva production, significantly shortened the time to saliva production, and greatly increased the amount of tear production relative to the control mice when examined about eight weeks after the usual time of disease onset.

The next day, we harvested their salivary and lachrymal glands, processed them for H and E staining, and determined the number of lymphocytic foci in the submandibular gland (SMG) and exorbital lacrimal gland (ELG) of each group. We observed no clear differences between the number of lymphocytic foci in the SMG of control and GABA-treated mice (Figure 1D). Similarly, the number of lymphocytic foci in the ELG of control and GABA-treated mice were similar (Figure 1F). The mean area of the infiltration was larger in the SMG and ELG of GABA-treated mice relative to the controls (Figure 1E,G), but this was not statistically significant.

### 3.2. Interventive GABA Therapy after Disease Onset in NOD.B10-H2^b^ Mice

**Figure 2:**
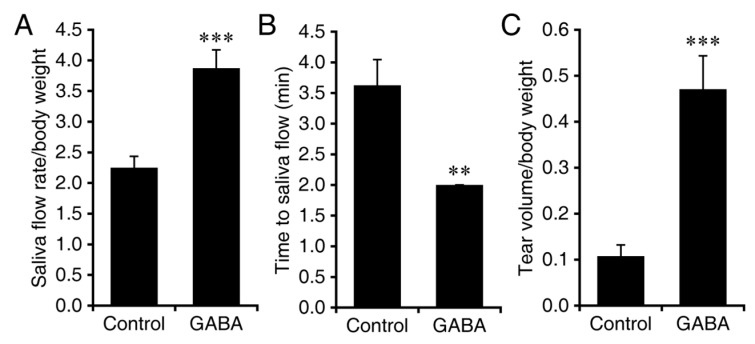
GABA treatment after the onset of symptoms preserves exocrine functions in NOD.B10-H2^b^ mice. Twenty-four-week-old NOD.B10-H2^b^ mice were placed on plain water (control) or water containing GABA (20 mg/mL) for six weeks. At 30 weeks in age, we analyzed their saliva flow and basal tear production. (**A**) Data shown are mean amount of saliva collected ± SD over 15 min post-isoproterenol and pilocarpine injection per gram body weight. (**B**) Time to saliva production (minutes) following isoproterenol and pilocarpine injection. (**C**) Tear production. Data shown are mean ± SD length of thread wetting (mm) over 20 s (average of both eyes) per gram body weight at 10 min post-pilocarpine and isoproterenol. *n* = 8 control and 9 GABA-treated mice. ** *p* < 0.01 and *** *p* < 0.001 by Student’s *t*-test.

In the second study with NOD.B10-H2^b^ mice, GABA treatment was initiated at 24-weeks in age, after the appearance of overt clinical symptoms. Twenty-four-week-old female mice were randomized to continuously receive plain water or water containing GABA (20 mg/mL). At 30 weeks in age, we analyzed their saliva flow and tear production. We observed that GABA-treated NOD.B10-H2^b^ mice produced on an average of 72% more saliva than those given plain water (*p* < 0.001, Figure 2A). The time to saliva production was also significantly shorter in GABA-treated mice (*p* < 0.01, Figure 2B). The amount of tear production was on an average about four-fold higher in the GABA-treated group vs. the plain water treated group (*p* < 0.001, Figure 2C). Thus, GABA treatment after the clinical onset of the disease helped preserve saliva and tear production, the most salient symptoms of SS.

**Figure 3:**
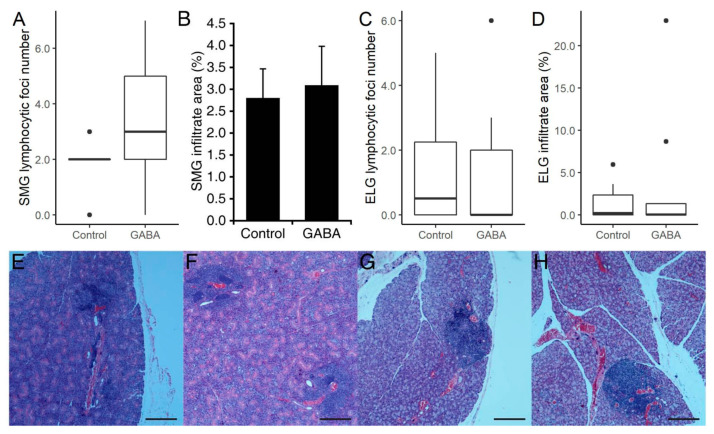
Number of foci and area of infiltrates in SMG and ELG from NOD.B10-H2^b^ mice treated with GABA post-disease onset. (**A**) Mean number of lymphocytic foci in the SMG, (**B**) mean area of infiltrates in the SMG, (**C**) mean number of foci in the ELG, and (**D**) mean area of infiltrates in the ELG of control and GABA-treated mice. For SMG *n* = 8 control and 9 GABA-treated mice. For ELG *n* = 4 control mice and 5 GABA-treated mice. There was no significant difference between groups by Student’s *t*-test (panel **B**) or by Mann–Whitney U test (panel **A**). Representative H and E-stained images from (**E**) control SMG, (**F**) GABA-treated SMG, (**G**) control ELG and (**H**) GABA-treated ELG. Scale bars are 250 μm. Non-normally distributed data are shown in boxplots with the borders of the box indicating 1st and 3rd quartile, the bolded line indicating the median, and with outliers plotted outside the whiskers and indicated by a “●”.

The next day, we harvested their salivary and lachrymal glands, processed them for H and E staining, and determined the mean number of lymphocytic foci and the area of infiltrates in each group. We observed some differences in the number of infiltrate foci and their area in GABA-treated vs. control mice, however, none of these differences were statistically significant (Figure 3). Thus, the GABA-mediated preservation of exocrine function was not associated with significant changes in lymphocytic foci number or area in the SMG or ELG.

### 3.3. Prophylactic GABA Treatment in C57BL/6.NOD-Aec1Aec2 Mice

To further test whether GABA has therapeutic potential for SS treatment, we performed similar studies in the C57BL/6.NOD-Aec1Aec2 mouse model of SS. In these mice, infiltrates become evident in exocrine glands beginning around eight weeks of age and these mice display pronounced salivary and lacrimal secretory dysfunctions by 16 weeks in age [13,16,48,49,51].

**Figure 4:**
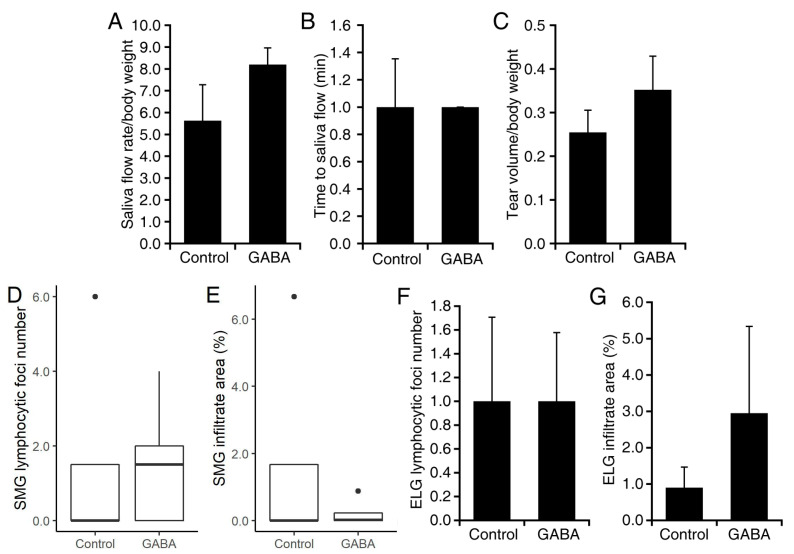
Measures of exocrine function in C57BL/6.NOD-Aec1Aec2 mice which received GABA prophylactically. (**A**) Saliva production. (**B**) Time to saliva flow (minutes) following isoproterenol and pilocarpine injection. (**C**) Tear production. (**D**) Mean number of lymphocytic foci in SMG and (**E**) mean area of infiltrates in SMG. For panels A-E *n* = 4 mice/group. (**F**) Mean number of foci in ELG, and (**G**) mean area of infiltrates in ELG of control and GABA-treated mice. For panels **A**–**E** *n* = 4 mice/group, and for panels **F**,**G** *n* = 4 control and 3 GABA-treated mice. None of the differences were significant by Student’s *t*-test if applicable. Non-normally distributed data are shown in boxplots with the borders of the box indicating 1st and 3rd quartile, the bolded line indicating the median, and with outliers plotted outside the whiskers and indicated by a “●”.

To test GABA as a prophylactic therapy, we randomized nine weeks old female C57BL/6.NOD-Aec1Aec2 mice to receive plain water or GABA (20 mg/mL) continuously for seven weeks. At 16 weeks in age, the mean saliva production in GABA treated mice was 46% greater than that in plain water treated mice, although this was not statistically significant (Figure 4A), and there was essentially no difference in the time to saliva production between these groups (Figure 4B). The volume of tears secreted by the GABA-treated mice was 38% greater than that of plain water-treated mice, although this too was not statistically significant (Figure 4C). Finally, there were no significant differences in the number of SMG and ELG lymphocytic foci or area between experimental and control groups (Figure 4D–G).

### 3.4. GABA Treatment after Disease Onset in C57BL/6.NOD-Aec1Aec2 Mice

**Figure 5:**
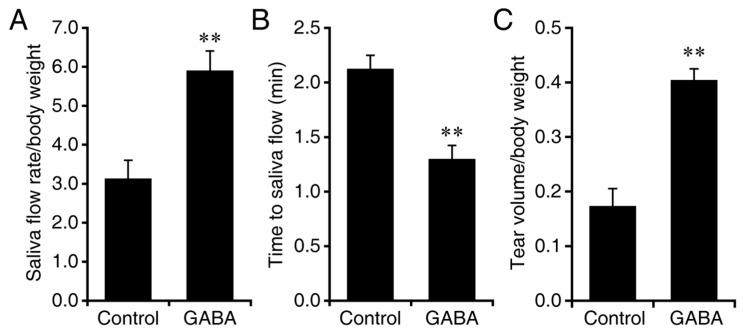
GABA administration after overt disease onset in C57BL/6.NOD-Aec1Aec2 mice preserves exocrine functions. Sixteen-week-old C57BL/6.NOD-Aec1Aec2 mice were placed on plain water or water containing GABA (20 mg/mL). At 20 weeks in age, we analyzed their exocrine function. (**A**) Saliva production. The data shown are the mean amount of saliva produced per gram body weight. (**B**) Tear production. The data shown are the mean length of wetted thread per gram body weight. (**C**). Time to saliva production. *n* = 4 control and 5 GABA-treated mice. ** *p* < 0.01 by Student’s *t*-test.

**Figure 6:**
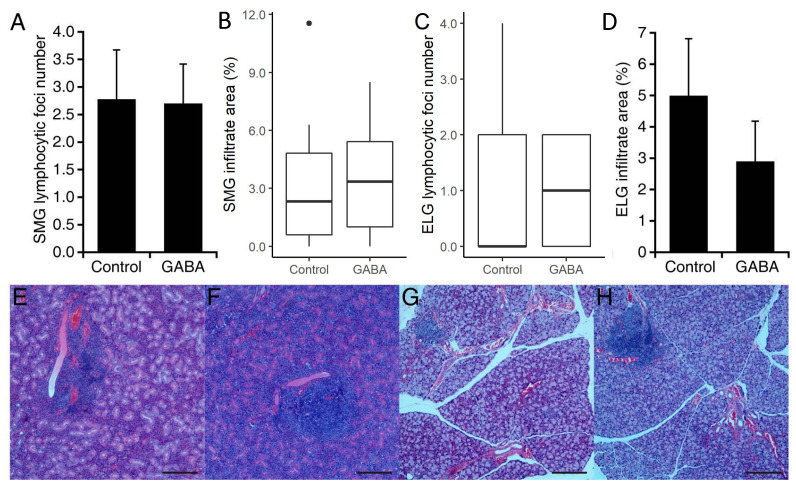
Number of foci and area of infiltrates in SMG and ELG from C57BL/6.NOD-Aec1Aec2 mice treated with GABA post-disease onset. The SMG and ELG from C57BL/6.NOD-Aec1Aec2 mice that were given plain water or GABA at 16 weeks in age were harvested at 20–24 weeks in age. (**A**) Mean number of lymphocytic foci in SMG and (**B**) mean area of infiltrates in SMG (*n* = 9–10 mice/group). (**C**) Mean number of foci in ELG, and (**D**) mean area of infiltrates in ELG in control and GABA-treated mice (*n* = 4 and 8 mice, respectively). Representative images of H and E-stained sections from (**E**) control SMG, (**F**) GABA-treated SMG, (**G**) control ELG, and (**H**) GABA-treated ELG. Scale bars are 250 μm. Non-normally distributed data are shown in boxplots with the borders of the box indicating 1st and 3rd quartile, the bolded line indicating the median, and with outliers plotted outside the whiskers and indicated by a “●”.

In a second study with C57BL/6.NOD-Aec1Aec2 mice, the mice were randomized to receive plain water or constant GABA treatment beginning at 16 weeks of age, after the onset of overt disease. These mice were subsequently examined at 20 weeks in age. We found that C57BL/6.NOD-Aec1Aec2 mice which received GABA mice produced an average of 88% more saliva than the control mice given plain water (*p* < 0.01, Figure 5A). Their time to saliva production was also significantly shorter in GABA-treated C57BL/6.NOD-Aec1Aec2 mice (*p* < 0.01, Figure 5B). GABA treatment also led to an average of 133% more tear production (*p* < 0.01, Figure 5C). Paralleling our observations in NOD.B10-H2^b^ mice, we observed no significant difference in the number or area of lymphocytic foci in the SMGs or ELGs of GABA treated vs. plain water-treated C57BL/6.NOD-Aec1Aec2 mice (Group data in Figure 6A–D), and representative images in Figure 6E–H).

## 4. Discussion

Currently, there are no effective therapies to slow the course of SS. SS treatment usually involves a combination of symptom management strategies which may be needed life-long. Given the beneficial effects of GABA treatment in mouse models of T1D, multiple sclerosis, and rheumatoid arthritis in mice, we asked whether this therapeutic approach could be extended to mouse models of SS. We studied GABA treatment in two different SS mouse models which occur in different genetic backgrounds: NOD.B10-H2^b^ and C57BL/6.NOD-Aec1Aec2 mice. These mice were given GABA prophylactically or after the appearance of overt symptoms. Weeks after initiating treatment, mice that received GABA treatment prophylactically had greater production of saliva and tears, although the higher levels were not always statistically significant relative to control mice that received plain water. When GABA treatment was initiated after the appearance of SS symptoms, the beneficial effects of GABA-treatment on exocrine functions were more evident, leading to significantly greater saliva flow and shorter time to saliva flow, as well as more tear flow in both SS models. Thus, our observations from two different models of mouse SS demonstrate that GABA treatment after the onset of overt disease ameliorates the key symptoms of SS.

Clinical studies with immunosuppressants such as Rituximab, Belimumab, and Abatacept failed to reach the primary endpoints of efficacy in SS patients (reviewed in [19]). These drugs target a specific immune cell population and may not have been effective because by the time SS becomes manifest there may be multiple immune cell populations involved in disease pathogenesis and targeting only one of these populations is insufficient to halt disease progression. Alternatively, these treatments may also target regulatory cells, such as regulatory B and T cells, and reduce their inhibitory activities leading to disease progression. As described above, GABA has a broad range of anti-inflammatory actions, including inhibiting Th17, Th1, and CD8^+^ T cell responses, modulating APC toward more anti-inflammatory phenotypes, enhancing Tregs, and reducing autoantibody levels, all of which may have contributed to its beneficial effects.

Histologically, we did not observe that the changes in the number and area of SMG or ELG lymphocytic foci were correlated with the improved exocrine functions in GABA-treated mice. Such observations are reminiscent of our past study of IL-27 treatment in C57BL/6.NOD-Aec1Aec2 mice, which also led to improved saliva flow rates without significant changes in SMG lymphocytic infiltrate numbers irrespective of the age at which treatment of the mice was initiated [53]. Indeed, although lymphocytic infiltrates in exocrine glands are important criteria for clinical disease, the extent of these infiltrations often do not correlate with disease severity in SS patients [54,55]. Interestingly, analysis of biopsied salivary glands from SS patients found that Th1 and Th17 infiltrates were largely outside the germinal centers, while Th2 and Tfh cells were localized within the germinal centers. [56]. Our studies have shown that GABA-R agonists inhibit Th1 and Th17 cells, but not Th2 cells [28,35,37], which might explain the persistence of large areas of lymphocytic infiltrates despite the much-improved saliva and tear production in GABA-treated mice. Additionally, studies of NOD mice noted that the severity of inflammation in the SMGs did not correlate with salivary gland dysfunction and saliva production tended to be greater in mice with a higher area of infiltrates [49]. Additionally, the number of ELG lymphocytic foci decreased and became more condensed with disease progression [57]—in this case, a GABA-mediated delay in disease progression would be expected to increase in the number and area of lymphocytic foci. Finally, in the T1D field, some experimental immunotherapies do not reduce the quantity of islet infiltrates, but rather shift their composition toward a “benign insulitis”. The benign insulitis is composed of more regulatory cells, such as Tregs, and fewer pro-inflammatory cells and is associated with long-term tolerance to the insulin-producing β-cells (e.g., [58,59,60]). In a similar fashion, GABA treatment may have led to a more “benign sialadenitis” in which pathogenic cells gave way to regulatory cells. It is also possible that GABA’s ability to inhibit inflammation may have allowed homeostatic acinar cell replication to gradually increase exocrine function. In future studies, we hope to assess whether these mechanisms, or others, contributed to our histological observations.

Oral GABA treatment has been tested in epilepsy patients for its ability to reduce seizures [61,62,63]. While it lacked clinical benefit, probably because GABA is unable to cross the blood-brain barrier, there were no adverse effects. A phase Ib GABA oral dosing study indicated that GABA is safe for consumption at up to 6 g/day [64] and a 2021 United States Pharmacopeia Safety Review of short-term GABA treatment found “no serious adverse events associated with GABA at intakes up to 18 g/d” [65]. Given the preserved exocrine function that we have observed in GABA-treated SS mouse models, GABA treatment appears to be a promising new approach to help ameliorate SS.

## 5. Conclusions

After the onset of overt SS, GABA-treatment led to significantly greater saliva flow and shorter time to saliva flow, as well as more tear flow in two different mouse models of SS.

## Data Availability

The data presented in this study are available on request from the corresponding author.
